# Preparation of Copolymer-Based Nanoparticles with Broad-Spectrum Antimicrobial Activity

**DOI:** 10.3390/polym9120717

**Published:** 2017-12-15

**Authors:** Yang Li, Pingxiong Cai, Zhang-fa Tong, Huining Xiao, Yuanfeng Pan

**Affiliations:** 1 Guangxi Key Lab of Petrochemical Resource Processing and Process Intensification Technology, School of Chemistry and Chemical Engineering, Guangxi University, Nanning 530004, China; liyang5854359@163.com (Y.L.); fg211d@gxu.edu.cn (Z.-f.T.); 2 College of Petroleum and Chemical Engineering, Qinzhou University, Qinzhou 535006, China; caipingxiong@qzhu.edu.cn; 3 Department of Chemical Engineering, University of New Brunswick, Fredericton, NB E3B 5A3, Canada; hxiao@unb.ca

**Keywords:** antimicrobial activity, nanoparticles, tailor-modified, guanidine-based, scanning electron microscope

## Abstract

Polyacrylate and guanidine-based nanoparticles which involve acrylate monomers and glycidyl methacrylate modified oligo-guanidine were prepared by a seeded semi-continuous emulsion polymerization. The results from transmission electron microscope and dynamic light scattering measurements showed that the nanoparticles were spherical in shape and the particle size was in the range of 80–130 nm. Antimicrobial experiments were performed with two types of bacteria, Gram-negative (*Escherichia coli*, ATCC 8739) and Gram-positive (*Staphylococcus aureus*, ATCC 6538). The as-synthesized cationic nanoparticles exhibited effective antimicrobial activities on *Escherichia coli* and *Staphylococcus aureus* with the minimal inhibitory concentrations at 8 μg/mL and 4 μg/mL, respectively. The mechanism of action of the resulted nanoparticles against these bacteria was revealed by the scanning electron microscopic observation. In addition, the films consisting of latex nanoparticles are non-leaching antimicrobial materials with excellent antimicrobial activity, which indicates the polymers could preserve their antimicrobial activity for long-term effectiveness.

## 1. Introduction

The infections caused by bacteria nowadays have a significant effect on the health of human beings. One of the great concerns is the robust or drug-resistant bacteria owing to extensive production and abuse of antibiotics [1]. Some typical and well-known bacteria called superbugs are methicillin-resistant *Staphylococcus aureus* (MRSA) and vancomycin-resistant Enterococcus (VRE) [2,3,4]. The demand for preserving appropriate facilities against microbial growth has become increasingly important for health care, various industries and everyday life [5]. Materials with properties of limiting or avoiding the development of bacteria are highly sought after in air conditioning equipment, freezers, filters, paints, toys, kitchen utensils, towels, paper and so on [6,7,8,9,10,11].

As one of the effective antimicrobial polymers, polyguanidines have attracted much research interest due to their high antimicrobial activity, hydrophilic property and effectiveness against a broad spectrum of bacteria [12]. The synthesis of guanidine-based polymers often relies on the condensation polymerization between the reactive guanidinium salts with appropriate structure and diamines [13]. The resulting antimicrobial polymers have been used in medical, fiber, textile, plastic, etc. [14,15,16,17]. However, the oligo-guanidine itself is highly water-soluble and possesses poor retention with substrates due to low molecular weight, thus limiting its use in the more demanding fields [18]. There is strong evidence that water soluble cationic polymers are more potent biocides than their low molecular weight analogues [5]; the star polymer containing guanidine chains even showed high antiviral activity [19]. Alternatively, the guanidine-based polymers have been covalently bonded with carrier or functional substrates via introducing vinyl groups. The modification of cellulose fibers via grafting copolymerization has been well received though the relatively low grafting efficiency remains as a key challenge [18,20,21]. 

In principle, the compact cell wall in bacteria consists of polysaccharides, proteins and nucleic acids, which prevent their colonies from being attacked by antimicrobial agents or drugs. Deteriorating the bacterial cell wall using nanoparticles represents one of the novel technologies developed recently in addressing the invasive effect of bacteria on a living cell [22,23,24,25,26]. In terms of synthetic approaches, several approaches have been developed in the past decade on fabricating the nanoparticles, such as self-assembly of polymers, seeded-emulsion polymerization, electrospinning and spray drying. The various materials as substrates could be rendered antimicrobial by such nanoparticles, leading to various applications in food packaging and healthcare areas, as well as textiles and water purification [27,28,29,30].

As an inexpensive, simple, high performance and effective synthetic method for the preparation of cationic emulsion or nanoparticles, the semi-continuous emulsion polymerization has been widely adopted due to its various advantages, such as avoidance of implosion and gelling, effective prevention of large latex particle formation and increase of the mechanical properties of emulsion membrane as well as the stability of the emulsion system [31,32]. In the current work, we aimed at developing non-leaching materials with permanent or, at least, for materials with life-term biocidal/biostatic activity via cationic emulsion polymerization. The glycidyl methacrylate -modified polyhexamethylene guanidine hydrochloride (GPHGH) was incorporated into acrylic latex using a two-step seeded semi-continuous polymerization strategy. The synthesized latex nanoparticles were characterized with Fourier transform infrared spectroscopy (FT-IR), dynamic light scattering (DLS) analysis and transmission electron microscopy (TEM). The efficacy of antimicrobial latex in inhibiting different pathogenic bacteria was assessed. The mechanism of action of the synthesized antimicrobial polymers against the microorganism was further revealed using a scanning electron microscope (SEM). The key objective of this work was to prepare inexpensive, high-performance and non-leaching latex with highly cationic-charged groups and small particle size for extended antibacterial applications, i.e., against both Gram-negative and Gram-positive bacteria.

## 2. Materials and Methods

### 2.1. Materials

Analytical-reagent butyl acrylate (BA), and butyl methacrylate (BMA) were purchased from Shanghai Aladdin Bio-chem Technology Co., Ltd. (Shanghai, China), washed with alkali solution prior to use. The initiator ammonium persulfate (APS) and the cationic emulsifier cetyltrimethylammonium chloride (CTAC), used in preparing the cationic nanoparticles or latex via emulsion polymerization, were purchased from Xi Long Chemical Technology Co., Ltd. (Guangzhou, China) and used as received without further purification. The crosslinker, ethylene glycol dimethacrylate (EGDMA), was obtained from Aladdin.

Polyhexamethylene guanidine hydrochloride (PHGH) was prepared by the condensation polymerization of hexamethylenediamine and guanidine hydrochloride which were purchased from Aladdin. Glycidyl methacrylate (GMA) was also supplied by Aladdin, and used as received.

### 2.2. Preparation of Cationic Latex Nanoparticles

**Scheme 1:**
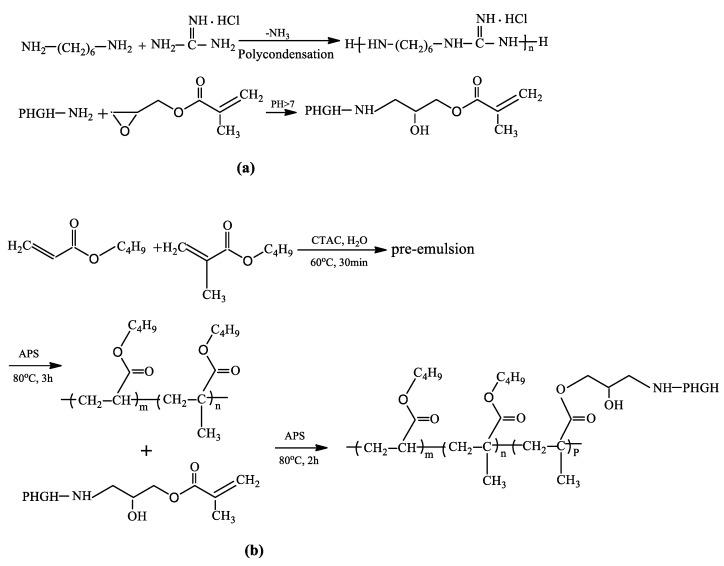
Schematic of GPHGH (**a**) and PA-co-Gs (**b**) syntheses.

The reactive vinyl groups were introduced by reacting PHGH with GMA to obtain GMA-modified PHGH (GPHGH); the schematic is showed in Scheme 1a. The molar ratio of glycidyl and amino groups was about 1.1:1. The reaction was carried out at room temperature in aqueous solution for 10 h [19]. The viscosity-average molecular weight (*M*_η_) of GPHGH was 1120 g/mol, measured using a viscometric method [33]. 

Then, a two-step seeded semi-batch emulsion polymerization was employed for constructing latex. A typical emulsion copolymerization process is described as follows. The mixture of 25 mL of deionized water, 30 mmol BA, 30 mmol BMA and 1 mmol surfactant CTAC (1.67% of the monomer moles) was first placed into a three-necked round-bottomed flask (100 mL) equipped with a mechanical stirrer, a reflux condenser and a feeding inlet. The system was heated to 60 °C and then purged with nitrogen for 30 min. Afterward, 0.04 g initiator APS (0.5 wt % of the monomer weight, dissolved in 5 mL of deionized water) was introduced into the flask. After that, the temperature was increased to 80 °C and maintained at that temperature under constant stirring at 200 rpm for 3 h in order to achieve a high conversion of the acrylate copolymer latex PBA-co-BMA or PA. Secondly, the amount of GPHGH and an aqueous solution of APS were placed in funnels and dropped into the above copolymer emulsion over 1 h at 80 °C. The initiator and GPHGH were also dissolved in distilled water, respectively, prior to being added. After the addition of the monomer and initiator, the emulsion polymerization was carried out at 80 °C for another 2 h. The resulting polymer is called PA-co-Gs latex (see Scheme 1b). The PA-co-Gs were purified through a dialysis tube with molecular weight cut-off 10,000 for 48 h, during which deionized water was changed for 3 times. Purified nanoparticles of PA-co-Gs were used in the entire work unless specified.

### 2.3. Characterization of Cationic Latex Nanoparticles

Fourier transform infrared spectroscopy (FT-IR) spectra were obtained with a Nicolet 5700 spectrophotometer (Thermo Fisher Scientific, Waltham, MA, USA) in the range from 4000 cm^−1^ to 400 cm^−1^. The hydrodynamic size and Zeta potentials of the resulting nanoparticles were determined by dynamic light scattering using a Zeta Sizer Nano Series (Brookhaven Instruments Corporation, Holtsville, NY, USA) with the concentration of nanoparticles at 1mg/mL in 0.1 mM NaCl aqueous solution at 25 °C. The reported average particle size values represent an average of three repeated measurements. 

The shape and size of the as synthesized latex nanoparticles were observed with Transmission Electron Microscopy (TEM), using TECNAI G2 20 TWIN (FEI, Hillsboro, OR, USA) instrument by drying a drop of the sample solution onto a copper grid covered with a conductive polymer.

The monomer conversion and solid content were determined gravimetrically. A certain quantity of emulsion (*m*_1_) was casted onto a Petri dish (*m*_0_), and dried to a constant weigh (*m*_2_) in a conventional oven at 110 °C. The solids content and final conversion were calculated by the following formulas, respectively:

$$\mathrm{solid}\;\mathrm{content}\;(\%)=\frac{m_{2}-m_{0}}{m_{1}}$$

$$\mathrm{monomer}\;\mathrm{conservation}(\%)=\frac{[m_{3}\times \mathrm{solid}\;\mathrm{content}(\%)]-m_{4}}{m_{5}}\times 100\%$$

where *m*_3_ is the total weight of all the materials put in the flask in each polymerization, *m*_4_ is the weight of emulsifier, and *m*_5_ is the weight of total monomers. 

### 2.4. Antimicrobial Activities of Cationic Latex Nanoparticles

Two methods used for the antibacterial activity evaluation in this work are broth microdilution and ring-diffusion. Both are typical assays and conducted in triplicate.

#### 2.4.1. Origin and Selection of Microbial Strains

The target microbes selected for the present study were *Escherichia coli* (*E. coli* ATCC 8739) as Gram-negative and *Staphylococcus aureus* (*S. aureus* ATCC 6538) as Gram-positive microbes, which were obtained from Guangzhou Jennio Biotech Co., Ltd. (Guangzhou, China) and maintained onto Mueller-Hinton agar (Merck, Darmstadt, Germany) favorable to their growth for 24 h at 37 °C.

#### 2.4.2. Preparation of the Inoculums

10 mL of bacterial suspension at proper concentration was discharged into a sterile phosphate buffer saline (PBS) solution for was homogenization. The initial concentration was set at 10^8^ CFU (colony forming units)/mL. After that, the suspension was diluted with sterile lysogeny broth (LB) to obtain an inoculum of 10^6^ CFU/mL on nutrient agar [34].

#### 2.4.3. Minimal Inhibitory Concentration (MIC) and Minimal Bactericidal Concentration (MBC)

Antimicrobial activities of the latex nanoparticles PA-co-Gs were evaluated by determining their minimum inhibitory concentrations (MIC) against the bacterial strains *E. coli* and *S. aureus* using a standard broth microdilution method [35]. The dispersions of PA-co-Gs were two-fold diluted (1024, 512, 256, 128, 64, 32, 16, 8, 4, 2 and 1 µg/mL) with LB. Then, a 0.20 mL from each of the diluted PA-co-Gs solutions was added to 1.80 mL of the nutrient broth in tubes containing each of the standardized bacterial suspensions (10^6^ CFU/mL), and seeded tubes were incubated in an incubator for 24 h at 37 °C. The test was conducted by observing the turbidity change. The change from transparent solution to turbid suspension is indicative of bacterial growth. The MIC was visually estimated from the lowest concentration of PA-co-Gs solution preventing visible growth of microorganisms. The diluted tubes without growth of microorganisms were plated onto the Mueller-Hinton agar (100 µL from each tube) and incubated at 37 °C for 24 h. This was used for determining the MBC which represented the lowest concentration of PA-co-Gs solution with no observable growth of microorganisms on the nutrient agar.

#### 2.4.4. Ring-Diffusion Method

The ring-diffusion method used in this work is a qualitative test to evaluate the antimicrobial properties and to confirm the non-leaching effect of the film samples. The concentration of latex was controlled at 10 wt %. Silicon wafers (approximately 10 mm × 10 mm) were selected as substrates, which were ultrasonically cleaned in acetone for 20 min prior to the deposition. The aqueous dispersion of nanoparticle was deposited on the surface of silicon wafer held in a petri dish; and dried in the air at room temperature to obtain polymer films after peeling. The films were carefully cut by punching to prepare film discs (about 6 mm in diameter) and put into culture dishes. In addition, a blank film disc was prepared from the acrylic latex without GPHGH. These film discs were sterilized immediately with ethanol and by exposing to UV radiation for 30 min before the experiments. The testing provided the visualized evidence for the potential diffusion or non-leaching of antimicrobial latex nanoparticles from the film in terms of the appearance or the absence of inhibition zone around the film samples [36,37]. 

### 2.5. Bacterial Morphology Revealed by SEM

Fresh *E. coli* and *S. aureus* bacterial strains were adjusted with LB broth to 10^6^ CFU/mL. Then, each bacterial suspension was diluted with the antimicrobial copolymer solution at concentration equal to 2× MIC and incubated for a period of 30 s. Then, the microbes were centrifuged (5000 rpm, 5 min), fixed with 20% glutaraldehyde solution for 30 min, washed with PBS, deionized water and ethanol/water mixtures at different ethanol concentrations prior to SEM imaging experiments. The prepared samples, coated with gold, were observed using a scanning electron microscope (SEM, S-3400N, Hitachi, Tokyo, Japan).

## 3. Results and Discussion

### 3.1. Influence of Monomer Ratios on Various Physical Properties of Latex Nanoparticles

Table 1 shows the influence of monomer ratios in syntheses on various physical properties of latex, including Zeta potential, mean particle size and conversion. Clearly, the conversion was decreased as the amount of hydrophilic GPHGH increasing, which is attributed to the steric hindrance structure of GPHGH. Whereas the mean particle size was increased, which indicated that the higher amount of GPHGH used, the larger the particle size, implying the occurrence of coalescence of monomer droplets during polymerization. Besides, all the latexes appeared to be stable due to high Zeta potential values (+40 mV or above); no phase separation of the emulsion was observed up to 6-month storage. 

### 3.2. FT-IR Analysis

**Figure 1:**
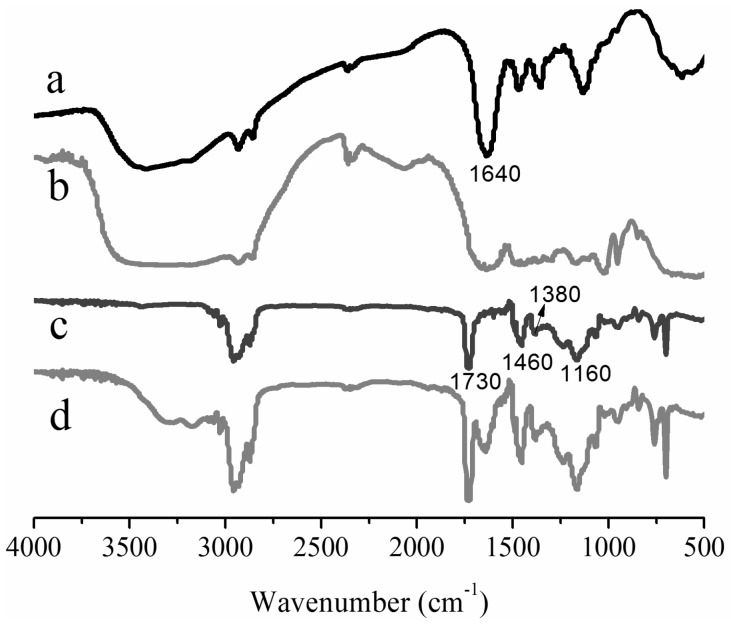
FT-IR spectra of (a) PHGH, (b) GPHGH, (c) PA and (d) PA-co-Gs.

Firstly, the pure acrylic latex without GPHGH was synthesized for the purpose of comparison with the PA-co-Gs latex. Figure 1 illustrates that all the polymers exhibited the characteristic stretching peaks of CH(CH_2_) at 2960 and 2874 cm^−1^, whereas the stretching vibration of C=O at 1730 cm^−1^ from acrylate groups were clearly visible for polymers PA (curve c) and PA-co-Gs (curve d) The carbonyl group in GPHGH (curve c) might overlap the characteristic peak of PHGH at 1640 cm^−1^ which was attributed to C=NH^+^ (N–H) stretching vibration and bending vibration [38]. The peaks at 1460 and 1380 cm^−1^ were caused by stretching vibration of CH_2_ groups. The disappearance of the characteristic absorption peaks at 1500 and 1250 cm^−^^1^ might be attributed to the reaction between primary amines in PHGH and epoxy groups in GMA, which also led to the broadening of the peak at 1640 cm^−1^ (see curve b). The result implied that PHGH reacted with GMA effectively. The disappearance of the characteristic absorption of C=C (1638 cm^−1^) in curve c and the new peak observed for the PA-co-Gs copolymer (curve d) at 1640 cm^−1^ (C=NH^+^) indicated that the monomers were indeed converted to the polymers. In other words, GPHGH was successfully introduced into the copolymer through the emulsion copolymerization.

### 3.3. TEM Image and Size Distribution of Latex Nanoparticles

**Figure 2:**
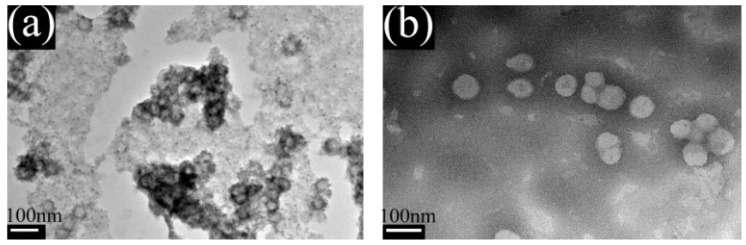
TEM images of PA-co-PGs before (**a**) and after (**b**) being purified.

The PA-co-Gs with the molar ratio of comonomers at 20:20:1 was used for TEM observation. Figure 2 shows a representative TEM image of PA-co-Gs before and after purification. Before the purification, PA-co-Gs latex particles were imbedded with unreacted monomers due to the limitation on achieving complete conversion. After purification, free monomers were removed and the PA-co-Gs latex particles appeared to be spherical in shape and well dispersed.

**Figure 3:**
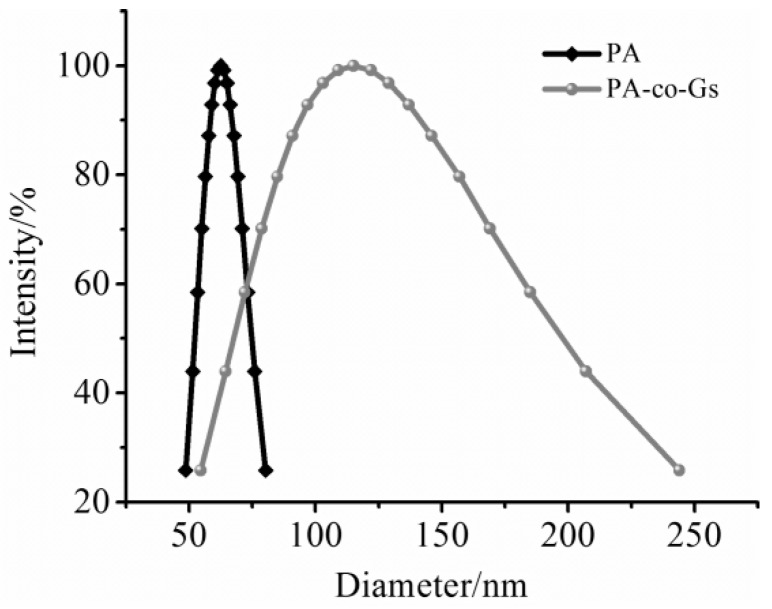
Particle size distribution of the PA and PA-co-Gs copolymer latex.

Figure 3 displays the size distribution of the PA and PA-co-Gs latex particles, obtained from dynamic laser light scattering measurement, for the same sample observed with TEM. The results of the size distribution indicated that the mean particle size of PA was about 60 nm with narrow distribution. In contrast, the major particle size of PA-co-Gs ranged from 80 nm to 130 nm. TEM image (shown in Figure 2b) also verified the final particle size within nanometer range. The representative diameter of PA-co-Gs latex particles were around 80 nm with well-dispersed regular spherical shape, which was in good accordance with the size distribution result obtained from DLS. However, the mean particle size of the latex particles measured with light scattering analyzer is larger than that obtained by the TEM test, which is due to the slight expansion of particles or the hydrodynamic layer around the nanoparticles when the latex particles were dispersed in the aqueous solution. Overall, the size variation of the copolymer latex is mainly determined by the size of the seeds (or cores), the amount of monomer for forming the shell and the amount of surfactant used in the seeded emulsion polymerization.

### 3.4. Antimicrobial Activity of Latex Nanoparticles

The minimum inhibitory concentration (MIC) and minimal bactericidal concentration (MBC) values of the latex nanoparticles with mixed monomers at different molar ratios, against both Gram-positive (*S. aureus*) and Gram-negative (*E. coli*) bacteria are summarized in Table 1. The purified nanoparticles showed MICs values ranging from 4 μg/mL to 16 μg/mL against gram-negative (*E. coli*) bacteria and from 1 μg/mL to 8 μg/mL against gram-positive (*S. aureus*) bacteria. The MBC values determined were in a range of 4–64 μg/mL for the purified nanoparticles. The MBC/MIC ratios were ≤4, showing that latex nanoparticles had a bactericidal effect against the mentioned bacterial strains [39]. Clearly, the purified nanoparticles were more effective against Gram-positive bacterium than Gram-negative bacterium. This behavior is attributed to structural difference between two types of bacteria. The additional membrane consisting of a phospholipid bilayer in Gram-negative bacteria often provides a barrier to prevent the attacking from pathogen-deactivating agents [3]. It is also noticeable in Table 1 that, in general, the enhancement in biocidal activity with the concentration of GPHGH increasing is partially due to the enhanced hydrophilicity after the incorporation of the GPHGH in the polycations, which has a positive effect on the antimicrobial activity. The cationic latex with the molar ratio of comonomers at 20:20:1 exhibited effective antibacterial activity with MIC as low as 4 μg/mL against *S. aureus* and 8 μg/mL against *E. coli*. Various influencing factors associated with the copolymer latex could affect their antimicrobial activity, including the polymer chain length, the sequence distribution of cationic chain segments, and the charge density of nanoparticles [40,41].

To further reveal or elucidate the mechanism of antimicrobial process involving the purified nanoparticles, the bacterial morphology of *E. coli* and *S. aureus* after the treatment with the copolymer PA-co-Gs latex nanoparticles for 30 s was examined by SEM and compared with the control sample.

**Figure 4:**
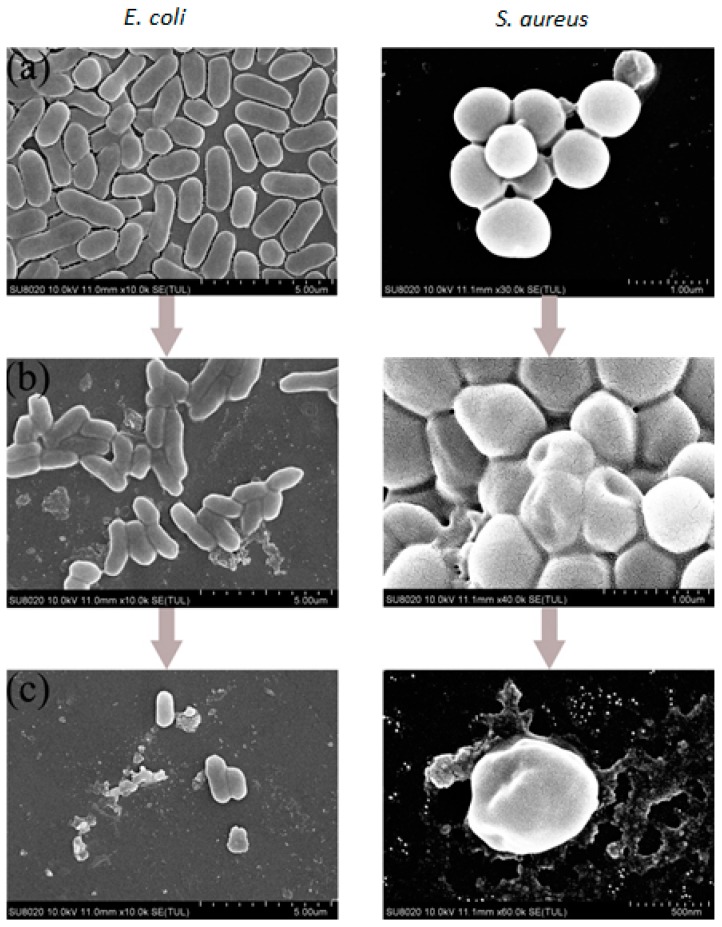
SEM images of the *E. coli* and *S. aureus* in the absence (**a**) and in the presence (**b**)/(**c**) of the PA-co-Gs nanoparticles (comonomer ratio: 20:20:1) after contact for 30 s.

Figure 4 demonstrates the significant changes in the cells after the treatment based on the SEM images. Clearly, the untreated bacterial cells have a well-defined shape with smooth surfaces (Figure 4a); whereas the cells treated with antimicrobial polymer show a remarkable change regardless of both Gram-negative *E. coli* and Gram-positive *S. aureus* ones. Aggregation, shrinkage and deformation in the bacterial wall were observed for the cells and cell lysis, which induces the leakage of intracellular material (Figure 4b,c). The results suggested that the bactericidal effect dominated the antimicrobial mechanism instead of inhibiting the growth of bacteria only, thus leading to the very efficient deactivation. Consequently, for cationic polymers, the mechanism of action is mainly attributed to the electrostatic interactions between bacterial membrane bearing anionic-charged phospholipids and cationic-charged nanoparticles. The deactivation is caused by the disrupting of cell membranes and the leakage of intracellular components.

### 3.5. The Antimicrobial Activity of the Latex Films

**Figure 5:**
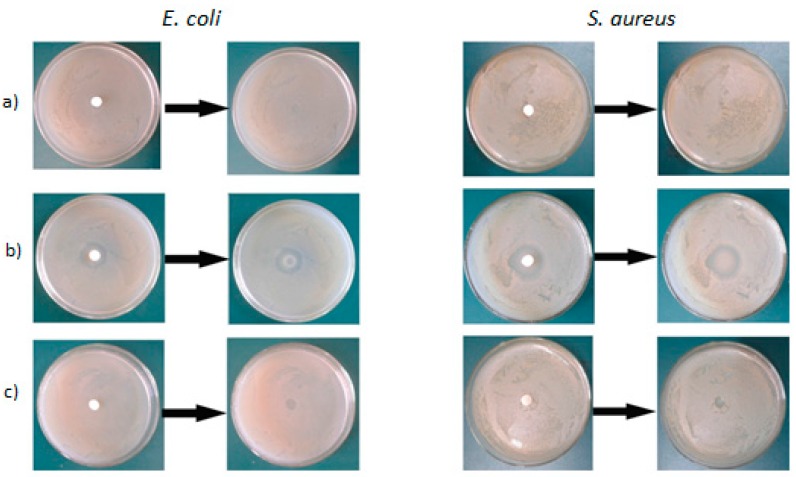
The inhibitory effect of PA (**a**), unpurified (**b**) and purified (**c**) PA-co-Gs films on the growth of *E. coli* and *S. aureus*.

The permanency of the antimicrobial copolymer was assessed by placing pieces of the films on the surface of agar plates previously inoculated with *E. coli* and *S. aureus*. The inhibitory effect of different film samples on the growth of bacteria is presented in Figure 5. As expected, pure acrylic latex film without GPHGH did not exhibit antimicrobial activity, therefore bacteria grew and the colonies completely covered the agar. For the antimicrobial latex samples before the purification, the inhibition zone is visible around the PA-co-Gs latex film on tested microorganisms. After the purification (removal of unreacted GPHGH), the inhibition zone around these pieces was hardly observed. The results indicated that the copolymer did not diffuse out of the film or the “leaching out” effect was eliminated, implying that GPHGH was covalently bonded in the latex nanoparticles. In other words, the films consisting of PA-co-Gs latex nanoparticles are non-leaching antimicrobial materials with excellent and durable antimicrobial activity.

## 4. Conclusions

Cationic nano-sized antimicrobial latex based on glycidyl methacrylate modified oligo-guanidine was successfully prepared via a feasible semi-continuous emulsion copolymerization. The formation of nanoparticles was confirmed with FT-IR, TEM and dynamic light scattering analyses. Dynamic light scattering results and TEM images showed that the formed copolymer latex or nanoparticles with molar ratio at 20:20:1 were well-dispersed with relatively uniform size ranging from 80 nm to 130 nm in diameter. The results obtained from the antimicrobial assay indicated that cationic antimicrobial latex nanoparticles exhibit a relatively high antibacterial activity against both Gram-positive and Gram-negative bacteria. From the experimental data, it can be concluded that the cationic antimicrobial latex nanoparticles have great potential to be used for versatile applications such as antimicrobial materials for biomedical and packaging applications.

## Figures and Tables

**Table 1:** Zeta potential, mean particle size and conversion of PA-co-Gs nanoparticles and MIC values of the nanoparticles against *E. coli* and *S. aureus*.

BA:BMA:GPHGH (mol/mol)	Conversion (wt %)	Zeta potential (+mV)	Mean particle size (nm)	MIC (μg/mL)		MBC (μg/mL)		MBC/MIC	
				*E. coli*	*S. aureus*	*E. coli*	*S. aureus*	*E. coli*	*S. aureus*
1:1:0	95.2	42 ± 1.5	56 ± 1	>1024	>1024	-	-	-	-
30:30:1	71.6	62 ± 1.6	81 ± 2	16	8	64	32	4	4
25:25:1	68.8	65 ± 1.7	88 ± 2	16	4	32	16	2	4
20:20:1	65.6	66 ± 1.8	93 ± 2	8	4	32	16	4	4
15:15:1	60.2	64 ± 1.7	97 ± 2	8	2	32	8	4	4
10:10:1	53.4	67 ± 1.9	113 ± 3	4	1	8	4	2	4
